# Coronary ostia obstruction after replacement of aortic valve prostesis

**DOI:** 10.1186/1746-1596-6-72

**Published:** 2011-08-02

**Authors:** Emanuela Turillazzi, Gabriele Di Giammarco, Margherita Neri, Stefania Bello, Irene Riezzo, Vittorio Fineschi

**Affiliations:** 1 Department of Forensic Pathology, University of Foggia, Ospedale Colonnello D'Avanzo, Via degli Aviatori 1, 71100 Foggia, Italy; 2 Institute of Cardiac Surgery, University of Chieti, Via dei Vestini, 29, 66100 Chieti, Italy

**Keywords:** aortic valve replacement, aortic stenosis, coronary ostia obstruction, myocardial infarction

## Abstract

Aortic valve replacement (AVR) is the gold standard for the treatment of severe symptomatic aortic stenosis. Complications directly related to surgical procedure are relatively infrequent. Coronary ostial stenosis is, generally, referred as late complication. Anecdotal reports concern coronary ostial stenosis as acute complication. A unique fatal case of intraoperative, bilateral coronary ostial obstruction by prosthetic valve leading to an extensive myocardial infarction is reported. Surgeons must have a high level of vigilance regarding the occurrence of acute myocardial ischemia and sudden death soon after AVR.

## Background

Aortic valve replacement (AVR) remains the gold standard for the treatment of severe symptomatic aortic stenosis. Late coronary ostial stenosis is described as late complication of the surgical procedure [1]. Anecdotal reports concern coronary ostial stenosis as acute complication: right ostial occlusion from aortotomy suture, ostial thrombosis as traumatic consequence from an aortic retractor, coronary artery spasm, calcium debris embolization and partial direct occlusion by the device or edematous reaction have been described [2-4].

A unique fatal case of intraoperative, bilateral coronary ostial obstruction by prosthetic valve leading to myocardial infarction is reported.

## Case presentation

### Clinical findings

A 50 -year old woman, previously submitted to surgical aortic valve replacement in 2003 for aortic valve stenosis was admitted to a cardiac surgery unit for replacement of dysfunctioning mechanical valve prosthesis. Echocardiographic evaluation documented a prosthetic dysfunction with the evidence of an increased peak gradient up to 105 mmHg and a prosthetic valve area by 0.58 cm^2 ^in a normal left ventricular function. Prosthetic valve replacement was then performed in August 2010. Cardiopulmonary bypass (CPB) was instituted between right atrium and ascending aorta and a moderate hypothermia was reached. Myocardial protection was achieved by retrograde injection of blood cardioplegia through coronary sinus as induction; it was completed by antegrade injection directly in both coronary ostia. After ascending aorta had been transversely opened, prosthetic dysfunction was evident as one of the hemidisks appeared to be locked by pannus ingrowth and fresh pivotal thrombi. No perivalvular leaks were described. The prosthesis was then removed; both coronary ostia were described quite close to the aortic annulus. Bovine pericardium bioprosthesis (Edwards Magna Ease 21 mm) was then implanted in supraannular position. Aortotomy was sutured using Teflon felt strips as reinforcement due to an extremely thin aortic wall and patient was uneventfully weaned from CPB. During skin suture patient experienced sudden severe hypotension; an ECG demonstrated signs of transmural myocardial ischemia. CPB was promptly reinstituted. Severe dilatation and hypokinesia of right ventricle and severe hypokinesia of interventricular septum were registered. Intraortic ballon pumping (IABP) was inserted through a femoral artery and a saphenous vein graft to right coronary artery (RCA) immediately added on a beating heart in the hypothesis of RCA ostial obstruction. At the end of the operation cardiopulmonary support by means of Extracorporeal Membrane Oxygenator (ECMO) through femoro-femoral access was initiated and IABP removed. Patient was then transferred to Intensive Care Unit (ICU) for postoperative course. Graft verification was not performed, either intraoperatively and postoperatively. Two days after, following progressive decrease of ECMO support along with a satisfying hemodinamical recovery, she was transferred to the operating theatre to remove mechanical assistance. One hour after the new admission to the ICU, cardiac arrest occurred pushing surgical team to reposition an emergent ECMO at the ICU bed. The day after patient was transferred again to the theatre to remove thrombi from left atrium evident at ecographic inspection. At that moment cardiac activity was silent in a very compromised systemic condition. Two days after the new admission to the ICU under a new femoro-femoral support she was declared dead.

### Pathological findings

**Figure 1:**
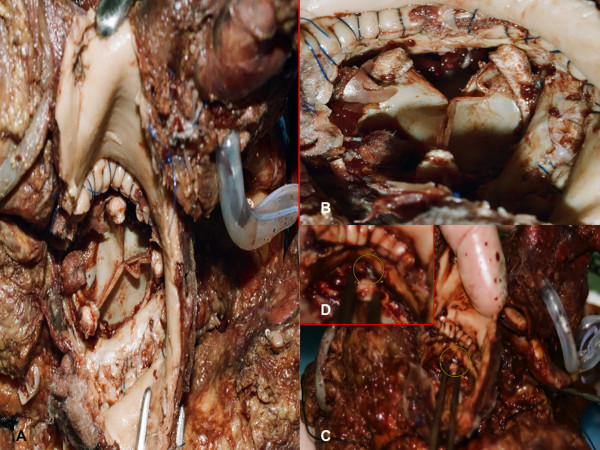
**Macroscopic findings**. (A) At the post-mortem inspection, a horizontal aortotomy was evident with prosthetic valve in situ after aorta was opened. (B) The coronary ostia are not visible. (C) A metal probe inserted into right coronary artery distally cut (insert in D), demonstrated the entrapment of the ostium in a stitch at the level of prosthetic annulus.

A complete post mortem examination was performed. Cerebral and pulmonary edema was detected. Cardiac size was mildly increased (11.5 × 10 × 7.7 cm), with conical shape; heart weight was increased (547g). At the inspection, a horizontal aortotomy was evident with prosthetic valve in situ after aorta was opened. Meticulous attention was devoted to observe the coronary ostia: the right one was not found at the inspection of inner surface of sinus of Valsalva. Using a metal probe inserted into right coronary artery distally cut, the entrapment of the ostium in a stitch at the level of prosthetic annulus became evident. Left coronary ostium was identified behind one of the bioprosthesis post that was responsible of its complete obstruction (Figure 1). Coronary vessels were fully patent during their course. Equally patent appeared to be saphenous graft to RCA. The inspection of both ventricles showed many foci of pale myocardium diffused to either free wall and interventricular septum.

**Figure 2:**
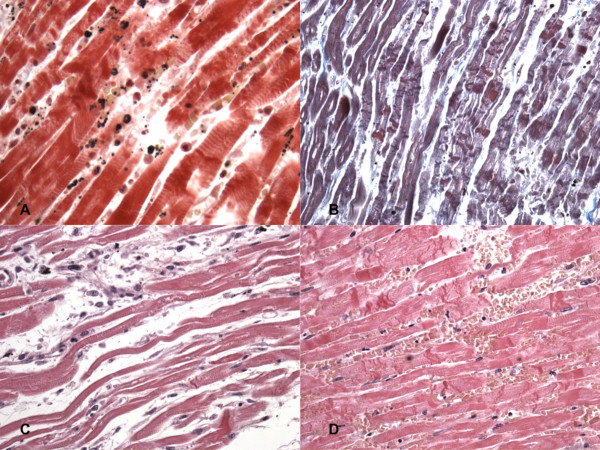
**Microscopic findings**. Acute myocardial infarction: polymorphonuclear leucocyte infiltration (A). Hypercontracted myocardial cells with markedly short sarcomeres and anomalous, extreme thickening of Z lines and rexis of the myofibrillar apparatus into cross-fibre, anomalous and irregular, up to a total granular disruption (B). Diffuse stretching of the myocardium with elongation of the sarcomeres and nuclei (C). Reflow areas (D).

Histological investigations showed haemostasis of all organs and mild cerebral and pulmonary edema. Specimens taken from the heart showed diffuse stretching of the myocardium with elongation of the sarcomeres and nuclei. Polymorphonuclear leucocyte infiltration was well evident at the periphery of the necrosis zones (Figure 2). Numerous foci of hypercontracted myocardial cells with markedly short sarcomeres and anomalous, extreme thickening of Z lines and rexis of the myofibrillar apparatus into cross-fibre, anomalous and irregular, up to a total granular disruption were also observed. Myocells pathological aspects were also studied on confocal laser scanning microscope: stretching of the myocardium in flaccid paralysis, resulting in a very early elongation of sarcomeres and nuclei; polymorphonuclear leucocyte infiltration was well evident.

Extramural coronary arteries showed nodular hyperplasia of smooth muscle cells and elastic tissue with fibrous replacement. Proteoglycan accumulation in the deep intima between tunica media and the fibrous cap was observed too. No critical stenosis was evident.

Final diagnosis of extensive myocardial infarction due to the intraoperative occlusion of both coronary ostia by bioprosthesis malpositioning was established as the cause of death.

## Conclusions

In the present case, extensive myocardial infarction due to the intraoperative occlusion of both coronary ostia by prosthetic valve was recorded as the cause of death. Since there are, to date, no reports of similar deaths in patients undergone to AVR, our report provides useful information on this complication of AVR.

Coronary ostial stenosis following AVR is believed to occur in 1% to 5% of AVR procedures. It is a life threatening complication which, generally, becomes evident from 1 to 6 months after the operation [5]. Several different mechanisms which include the possibility of microinjuries and local hyperplastic reaction related to the infusion pressure and/or low temperature of cardioplegic solution and overdilation of the vessel by the tip of the cardioplegic catheters are thought to be involved [1,5]. Other mechanisms were hypothesized such as widespread intimal thickening and fibrous proliferation in proximity of the aortic root, presumably as a reaction to turbulence around aortic ball valve prostheses. An immunological reaction to the heterograft after AVR has been considered in cases of bilateral ostial coronary arteries stenoses revealing several months following the surgical procedure [1,2]. On the basis of these evidences the exact mechanism underlying late coronary ostial stenosis following AVR is unclear.

Anecdotal reports describe the rare occurrence of acute coronary ostial stenosis; right ostial occlusion from aortotomy sutures and ostial post-traumatic thrombosis due to aortic retractor have been described [2]. In exceptional cases, embolism from debris, more often calcium related to aortic valve decalcification, or left atrial thrombectomy can be involved [1,2]. Coronary artery spasm has been recognized as a possible cause of hemodynamic and arrhythmic instability after aortic valve replacement [6]. Occasionally, secondary fibrosis in the area of suture placement may occur causing ostial stenosis [7]. Finally, use of surgical glue in aortic surgery, or compression from outside due to the glue used to protect the anastomosis may cause stenosis of one or both coronary ostia [8].

Conclusively, there is a reasonable body of evidence that acute coronary ostial stenoses may occur, even if rarely, after AVR and this complication may be life threatening if not promptly recognized, leading to myocardial ischemia, infarction, or fatal arrhythmia. Consequently, it is important to have an high index of diagnostic suspicion if circulatory collapse and/or signs of myocardial ischemia occur soon after surgery. Transesophageal echocardiography can be useful in diagnosis of acute complications of cardiac surgery; however urgent coronary angiography remains the gold diagnostic tool [8].

In our case, both coronary ostia were iatrogenically occluded by the prosthesis: the right one appeared to be entrapped by a stitch and the left one was occluded by prosthetic post. Neither intraoperative transesophageal echocardiography or postoperative coronary angiography were performed, so impeding to reach a prompt diagnosis.

From a clinical point of view, surgeons must have an high level of vigilance regarding the occurrence of acute myocardial ischemia soon after AVR and must be ready to perform either an intraoperative verification of patency or an early coronary angiography during ICU stay since these diagnostic tools may reveal mechanisms underlying ischemia which could make necessary a surgical approach (coronary stenting, device removal and/or re-replacement with a smaller valve size with or without annular enlargement) [3,8].

## Consent statement

Written informed consent was obtained from the Medical Examiner Department, Court of Justice, for publication of this case report and accompanying images. A copy of the written consent is available for review by the Editor-in-Chief of this journal.

## List of abbreviations

AVR: aortic valve replacement; CPB: cardiopulmonary bypass; IABP: intraortic ballon pumping; RCA: right coronary artery; ECMO: Extracorporeal Membrane Oxygenator; ICU: intensive care unit.

## Competing interests

The authors declare that they have no competing interests.

## Authors' contributions

SB drafted the manuscript. MN carried out the istological analysis. IR performed the microscopic analysis. ET, GDG and VF conceived of the study, and participated in its design and coordination and helped to draft the manuscript.

All authors read and approved the final manuscript.
